# Monthly Summary

**Published:** 1876-06

**Authors:** 


### MONTHLY SUMMARY.

---

*Bonwill's Method of Inducing Anæsthesia.*—At an introductory lecture delivered by Prof. Addinell Hewson, at the Pennsylvania Hospital, and reported in the Philadelphia *Medical Times*, of March 4th, by Dr. T. H. Bradford, a description was given, with illustrations, of Dr W. G. A. Bonwill's method of diminishing or allaying sensibility by rapid respirations. We quote the following portions of the article:

"You have, all of you, I have no doubt, experienced the effects of rapid and deep respirations after violent running, or of blowing hard to ignite a fire—especially the confusion of sight and bewilderment of mind. These Dr. Bonwill recognized many years ago, associated with numbness of sentiment nerves, as dependent on the rapidity of the respirations. Pursuing the subject, he has brought it to practical use in his profession—that of dentistry—in which he uses it constantly to diminish the sensitiveness of dentine, and even to produce such sensibility as to allow of the extraction of a molar tooth without pain. Of the latter I have had a demonstration in my own family, which has led me to the study of the subject myself, and this with the most gratifying results. I have used it in stitching wounds, in handling over-sensitive parts, and in probings and the like.

The method was tried upon two boys, who, on account of emotional excitement, failed to carry out the instructions in regard to breathing; and the writer of the report then volunteered to try the process before the class.

"It was his first attempt, and was made sitting erect, with his right hand resting upon a table. Breathing rapidly [for about three minutes] was attended first with a tingling sensation of the surface, especially of the fingers, and a feeling as though the surface was all swelling. Then there followed a dizziness or confusion in the head, with consciousness well preserved, but with a feeling of inability to resist or act in an independent way. He remembered well being frequently asked by the doctor if he was hurting him, but had no recollection afterwards of the pin sticking him, much less of its having been firmly imbedded in his flesh, as he found it when he had ceased the rapid respirations and the anæsthetic effect had passed off."

"Some cases of interest, as showing important points in diagnosis, were after this brought before the class, and the rest of the hour was occupied in their discussion. After dismissing the class, Dr. Hewson went with the writer to the receiving ward, where they found waiting, a boy who had fallen upon the ice an hour previously and sustained a severe injury to his left wrist. He was evidently suffering, and in great dread of being hurt, and the doctor directed him at once to try the rapid respirations. This, in two minutes and a half by the watch, showed some dizziness in the boy's head, when the doctor picked up the limb and moved it about with the utmost freedom, diagnosing a bad sprain of the wrist, and the absence of fracture. When the boy was recovering, he took to crying, on account, he said, of the dizziness and confusion he had experienced. Nothing could have been more satisfactory than this case in its results. He said positively *he was not* suffering any pain, the limb having been put in the easiest position possible."—*New Remedies.*

---

*The Arrest of Hemorrhage by Torsion.*—This method, popular with some surgeons in this country, was the subject of a la[illegible]e essay by Dr. Tillaux, of Paris, and read at the Sociète de Chirurgie, with these conclusions: 1. Torsion is applicable to arteries of every calibre, and more especially to large arteries.

2. A single forceps is required, whatever be the size of the artery. 3. The artery should be seized obliquely, and not in the course of its continuity, and in such a manner as to comprehend within the teeth of the forceps all the three tunics of the vessel in their entire breadth. 4. The torsion should be continued until the portion seized is entirely detached, 5. The thrusting back the tunics toward the heart, as advised by Amussat, and the limited torsion of Amussat and the English surgeons, are useless. 6. Torsion is applicable to atheromatous and inflamed arteries. It is a valuable means of arresting hemorrhage at the bottom of wounds. 7. It favors immediate union by the absence of a foreign body. 8. Like the ligature, it protects from primary hemorrhage. 9. It is a better protective against secondary hemorrhage than the ligature. Since 1871, M. Tillaux has exclusively adopted torsion, both in great and small operations ; and in about a hundred great operations in which he has employed it, he has never met with either primary or secondary hemorrhage.—*Med. and Surg. Reporter.*

---

*A Curious Case of Fracture.*—Every physician or surgeon during a long life of practice meets with many anomalous and sometimes amazing cases. The following, which took place some twenty years ago at Memphis, Tenn., is to the point :

Two local editors had a slight " unpleasantness" in the editorial rooms, when one gave the other a blow in the face, inflicting what was supposed to be a serious wound. I was hastily summoned, the messenger stating that Mr. R. had been struck in the face, fracturing his lower jaw, and that the ends of the bones could be felt " sticking out." I was with the sufferer in a few minutes [who, by the way, had thick, bushy whiskers,] and on examination found that what was supposed to be the ends of the bone previously felt through the whiskers was in reality the top end of a common cedar pencil protruding from the skin about two inches. It had penetrated the left side of the neck about two inches from the apex of pomum adamum, passing just below the hyoid bone. On withdrawing the pencil I found that it had penetrated four and a half inches, the point having grazed the left side of the spine. When removed the pencil was found to be six and a half inches long, with an unbroken point. No unpleasant symptoms followed. The reconciliation was as rapid as the cure.—*C. S. Fenner, in Louisville Med. News.*

---

*Female Medical Students.*—One of the weekly journals of St. Petersburg gives the following statistical particulars touching the number of women studying or practicing medicine in Russia :—During the year 1875 the number of female medical students amounted to 171, of whom 102 belong to the nobility, 17 to high trades, 14 to small trades, 12 to the clergy, and 24 to the different classes of the gentry. Among the number, 23 were Jews, 12 Armenians, and 3 Lutherans; the remainder were of the orthodox persuasion; 23 were married, and 53 had already received their diploma. Another Continental journal also gives some details of the number of female students in the University of Berlin. Their number is 30, of whom 2 are studying law, 25 medicine, and 3 philosophy.—*Med. and Surg. Reporter.*

---

*The Parasite Theory of Disease.*—The editor of the *Medical Press and Circular* says, on this much debated subject:—The idea that minute atmospheric germs were the cause of diseases, as well as putrefactive changes, is no new theory; it originated with Kircher and the pathologists of the seventeenth century. Since their time it has been frequently revived, and hundreds of more carefully conducted experiments than those made in their days have certainly done very little toward confirming it in the minds of the profession. The late Professor Hughes Bennett and his able assistants conclusively proved the fallacy of the theory; in fact, their numerous experiments indicate that the production of infusorial life depends, for the most part, upon temperature, chemical constitution, density, and other equally important physical properties of the air, rather than on living floating organisms.—*Med. & Surg. Reporter.*

---

*Skull Changes in Rickets.*—Not long since, at a society meeting in London, Dr. E. Bennett showed many specimens illustrating deformities due to the action of rickets, not to congenital causes. These deformities were: (1) inbending of the basilar portion of the occipital bone round the foramen magnum; (2) a diaphanous condition of the occipital bone; (3) a marked obliquity of the base of the skull; (4) great weight of the skull; and (5) the formation of a ring of bone round the articulating surfaces of the cervical vertebræ in elderly subjects. Craniologists should note these pathological changes, as they are at times deceptive.

---

*New Mode of Obtaining Local Anœsthesia.*—"M. Latamendi has found that when Richardson's apparatus is used, rubefaction and a sensation of cold is produced. If at this moment a slight incision of about half an inch is made with a curved bistoury, not deeper than the epidermis and the upper layer of the cutis from the incised spot an anæmic zone is formed which goes on spreading. If the ether spray is continued, the region becomes bloodless, and complete anæsthesia has been obtained. The knife cuts the part like butter, the spot resembling coagulated fat. Around this an annular patch is observed, which is not so completely anæmic as the centre. The spray directed upon this renders it also completely anæsthetic. Thus the anæmia can be extended in every direction, around and up the arm. If the spray be suspended, the effects disappear quickly ; but the spray being resumed, in few seconds the ischæmia returns."—*Lancet.*

---

*Morphia in Neuralgia.*—Mr. Spencer Thompson writes on the subject of neuralgia as follows :

Even phosphorus, we know, will, after a time, lose its power in some obstinate cases of neuralgia, at all events its power of rapid relief ; and then it is that the invaluable hypodermic administration of morphia comes to our aid. This remedy, and its mode of administration, are too well known to require comment here ; but it is far from being as generally employed as it ought to be. This, perhaps, is due to various causes, but of these I believe the principal are, the means of administration not being always readily available, and the objection of patients to the pain consequent upon the use of coarsely constructed instruments. The first of these objections I have endeavored to meet by the use of a very portable hypodermic apparatus, enclosed in a metallic case, with ample supply of needless, and the great desideratum, an always moist and efficient piston ; and be always carrying a supply of gelatine discs. The second objection is met by the use of very fine steel needles only. The discs which contain one sixth of a grain in each, are a very safe and efficient dose for most cases, although in some it may be well to begin with a less amount, and in many it may be advisable to increase the dose considerably, half a grain, or even double that amount. I may here give it as the result of a very large experience in the hypodermic administration of morphia, that concentrated solutions are the reverse of advantageous. In the first place, they are not so safe as the more dilute ; and, in the second, they do not act so quickly and agreeably. The usual strength I employ is one grain of hydrochlorate of morphia in forty minims of water, rarely in thirty. The slight increase of bulk is of no consequence, and in administrations I can count by the thousand, I have never seen the slightest bad consequence, in the way of abscess or otherwise, result to the patient.—*Lancet.*

---

*Salicylic Acid for Offensiveness of Breath and Expectoration.*—Dr. Da Costa, prescribes salicylic acid, five grains, dissolved by means of a drachm of glycerine in half an ounce of water taken three times a day, in cases where the breath or expectoration are offensive. If internal administration does not accomplish the desired result, it can be used with the atomizer in a solution of similar strength.—*Med. and Surg. Reporter.*

---

*Food for Lean Women.*—If any one wishes to grow fleshy, a pint of milk taken before retiring at night will cover the scrawniest bones. Although now-a-days we see a great many fleshy females, yet there are many lean and lank ones who sigh for the fashionable measure of plumpness, and who would be vastly improved in health and appearance could their figure be rounded with good solid flesh. Nothing is more coveted by thin women than a figure, and nothing else will so arouse the ira and provoke the scandal of one of the " clipper builds " as the consciousness of plumpness in a rival. In cases of fever and summer complaint, milk is now given with excellent results. The idea that milk is feverish has exploded, and it is now the physician's great reliance in bringing through typhoid patient's or those in too low a state to be nourished by solid food. It is a great mistake to scrimp the milkpitcher.—*Druggists Circular.*

---

*Cooking by Cold.*—It is a curious fact, not generally known that the action of intense cold on arganic substances is similar to that of a higher degree of heat, and that, when subjected to a very low temperature, meat can be brought to a condition similar to its state when cooked by actual warmth. Quite recently a Hungarian chemist, Dr. von Sawiczewsky, who, it appears, has investigated all the various ways suggested for preserving meat [by chemicals, cooking by heat and hermetically sealing, etc.,] and has found points of objection to all, has attempted the preparation of the material by subjecting it in a perfectly fresh state to a tempature of 33° below zero, F., and sealing it afterwards in tins The results obtained have been highly satisfactory ; the meat on being removed from the cans appears, in point of smell and color, as fresh as if just taken from the butcher's stall. Although partially cooked, and thus requiring less fuel to complete its preparation for the table, it is entirely without the taste of meat which has been partially subjected to any heating process, and may be roasted, boiled or otherwise treated, the same as if it were fresh. A commission appointed by the German governments has lately conducted a series of careful and successful experiments upon the process ; and as a final test two corvetts of the German navy, being about to circumnavigate the globe, have been supplied with a large stock. An extensive factory is being erected in Hungary for its manufacture.—*Scientific American.*

*Hardening Compound for Steel—*

| | |
|---|---|
| Powd. borax, - - - - . - - | 3 parts. |
| Sal-ammoniac, - - - - - - - | 2 " |
| Bone dust, - - - - - - - | 1 part. |

Mix. Heat the steel red hot, shake a small quantity of the compound over it ; place it again in the fire till the composition smokes on the steel, which will be much below the ordinary welding heat, and then hammer it, Properly done, it gives a first-class hard steel.—*Druggists Circular.*

---

*Radical Cure for Piles.*—Dr. A. B. Bowen, of Magnoketa, Iowa, writes . " In a recent number of *The Record*, my attention was directed to the treatment for *nœvus* by hypodermic injection. From the similarity of the anatomical structure of the nœvus to hemorrhoidal tumors, I was induced to try the remedy. In the latter I used carbolic acid and ergot [fl. ext.] in equal parts, injecting from ten to fifteen minims of the solution into the spongy, vascular hemorrhoidal tumor. This was repeated about once a week for five or six times, when the tumor had entirely disappeared. I have tried this in several cases, and it acts like a specific."—*N. Y. Med. Record.*

---

*Painful Points in Neuralgia.*—More than a score of years ago, M. Valleix, the distinguished author of the *Guide de Medicin Practicien*, called attention to the frequency of vertebral *points douloureux* in neuralgia, and the necessity of directing the treatment to them. Prof. Duplouy, of Bordeaux, now publishes the particulars of three cases of neuralgia that resisted all other treatment, and eventually rapidly subsided after the application of blisters to these points.—*Med. and Surg. Reporter.*

---

*Jaboraudi in Colds.*—A French writer suggests that we are now in possession of a medicine which is destined to render signal service in colds. It is jaborandi, which, by its sudorific properties, and by its exciting action on the glands of the air passages and salivary glands, combines the principal pharmacodynamic effects that have heretofore been sought from various medicines. An infusion of one drachm of the leaves in a small quantity of water constitutes an efficient means against colds, if taken early.—*Ibid.*

---

*Torsion of Arteries.*—Since 1871, M. Tillaux has not ligated the arteries in his operations, and in over one hundred major operations, in which he has employed torsion, he has not had a single case either of primary or of secondary hemorrhage. In a memoir on this subject, read before the *Societe de Chirurgie*, he draws the following conclusions :

1. Torsion is applicable to all arteries, and especially to the large ones.
2. Only one pair of forceps is required for the operation.
3. The artery should be seized obliquely, and not perpendicularly to its axis.
4. The torsion should be continued untill the end seized by the forceps is detached.
5. It is useless to limit the torsion ; it confines itself to the portion seized, or, at most, to one or two centimetres beyond this.
6. Atheroma is no counter-indication to torsion.
7. Torsion favors immediate union.
8. Torsion prevents primary hemorrhage as well as the ligature does.
9. Torsion presents a better safe-guard against secondary hemorrhage than the ligature.—*La France Medicale.*

---

*The Exposure of Spiritualism.*—A public sèance was recently given at Chickering Hall by Mr. W. Irving Bishop, for the purpose of exposing some of the impositions connected with spiritualism. His hands were bound behind him, and while seated in a chair were tied to a ring in a fixed upright. His head was secured to the same upright by a piece of muslin encircling his neck, and his feet were tied together. While thus secured he rang bells, played on the guitar, drove nails into a board, covered his head with an inverted pail, and performed behind a screen other astonishing feats. The screen being removed the same performances were repeated. The length of hand, flexibility of wrist and trunk, and the looseness of the skin of the wrist, enabled the performer to accomplish these wonders without a great deal of effort, and despite the tight bandages. Notwithstanding his limited opportunity for movement he could bring his hands sufficiently forward, by sliding the skin of the wrist, to manipulate with comparative ease. The performances were under the auspices of the St. John's Guild, and were well patronized. Dr. W. A. Hammond opened the entertainment with an address on the history of humbuggery. He traced it from the dark ages down to the present, giving illustrations of the gullibility of certain confiding individuals, and concluded with an allusion to the differences of opinion among neurologists concerning the value of the ophthalmoscope in the detection of brain disease.—*Med. Record.*

*Gelseminum Sempervirens in Facial Neuralgia.*—Dr. Spencer Thompson calls special attention to the value of tincture of gelseminum for the relief of neuralgic pain. According to his experience, the remedial power of gelseminum "seems confined to those branches of the trifacial nerve supplying the upper and lower jaws—more particularly the latter, and more especially when in either jaw the pain is most directly referred to the teeth or alveoli; indeed, he can scarcely recall an instance of the above in which relief was not speedily and thoroughly given." Dr. Thompson now almost invariably prescribes for an adult twenty minims of the tincture as a first dose, to be repeated any time after an hour and a half, if relief is not given. He has rarely had to order a third dose in any of his 40 cases, and he has never found any inconvenience result from the dose. In one instance, a gentlemen unadvisedly took thirty minims of the tincture at once, and immediately afterwards went out driving; he told Dr. T. that he experienced for an hour or two some uncertainty of vision when guiding his horse. A severe attack of neuralgia of the jaw was, however, cured by the one dose, and did not return.—*London Lancet.*

---

*A Lotion for Fœtid Feet and Arm-Pits.*—

| | |
|---|---|
| Bromo-Chloralum, - - - | 1 part. |
| Water - - - - - | 15 parts. |

Bathe three or four times a day. For these and other similar cases, this formula has proven very efficacious. In many instances it has been successful when all others have failed.—*Journal of Materia Medica.*

---

*Sensitive Artificial Eyes.*—In 1873, Mr. May, a telegraph clerk employed at the Valentia station of the Atlantic cable line, first observed that the electrical resistance of selenium was instantly altered by light, the resistance being diminished by increase of light.

Dr. Siemens has made use of this peculiarity of selenium in the construction of an artificial eye. An electrical circuit is arranged, of which a bit of selenium forms a part, and constitutes the retina. When a strong light is admitted into the lens and falls upon the selenium retina, the current of electricity flows, and is made to work the artificial lids of the eye, opening or closing them according to the intensity of the light.—*Med. and Surg. Reporter.*

*Cure for Corns.*—A correspondent of the *Scientific American* writes as follows, to one who asks for a remedy for corns :—Bind raw cotton on your corn at night before going to bed, and then saturate the cotton with spirits turpentine. It will remove the most obstinate corn either hard or soft, in four or five applications. The skin will be apt to peel off the toe, but this is rather an advantage, as it helps to remove the corn.

---

*A Large Mouth.*—Dr. Pixton, dentist, of Lancaster, has the impression of the mouth of a colored woman, taken by Dr. Robinson, of Rome, New York. The jaw is three inches from front to back, and three inches across, constituting the largest human mouth known to the dental profession in this country. The ordinary mouth is only 1½ by 2 inches.—*Medical and Surg. Journal.*

---

*Graduates in Dentistry.*—Only one hundred and forty six gentlemen graduated from the different Dental Colleges during the recent commencement season.—*Med. Record.*

---

*Johns Hopkins Hospital.*—It is proposed to make the medical department of this institution independent of students' fees. —*Exchange.*

---

*Eucalyptus in Ague.*—Dr. Paul Boyce says, in the Virginia *Medical Monthly* :—A case of ague and fever, which had been treated with quinia, arsenic, etc., was cured in four days by simply taken the essence of eucalyptus (prepared from the oil distilled from the leaves) ʒij three times a day. I believe we possess in the eucalyptus a remedy not inferior to the cinchona alkaloids. The oil applied to the nerve of a tooth soon destroys its sensitiveness and quiets the pain. In purulent catarrhal affections of the urethra *it acts like a charm.*—*Med. and Surg. Reporter.*

